# Systems analysis of gene ontology and biological pathways involved in post-myocardial infarction responses

**DOI:** 10.1186/1471-2164-16-S7-S18

**Published:** 2015-06-11

**Authors:** Nguyen T Nguyen, Merry L Lindsey, Yu-Fang Jin

**Affiliations:** 1 Department of Electrical and Computer Engineering, University of Texas at San Antonio, San Antonio, TX 78249, USA; 2 San Antonio Cardiovascular Proteomics Center, University of Texas Health Science Center at San Antonio, San Antonio, TX 78229, USA; 3 Mississippi Center for Heart Research, University of Mississippi Medical Center, Jackson, MS 39216, USA; 4 Research Service, G.V. (Sonny) Montgomery Veterans Affairs Medical Center, Jackson, MS 39216, USA

## Abstract

**Background:**

Pathway analysis has been widely used to gain insight into essential mechanisms of the response to myocardial infarction (MI). Currently, there exist multiple pathway databases that organize molecular datasets and manually curate pathway maps for biological interpretation at varying forms of organization. However, inconsistencies among different databases in pathway descriptions, frequently due to conflicting results in the literature, can generate incorrect interpretations. Furthermore, although pathway analysis software provides detailed images of interactions among molecules, it does not exhibit how pathways interact with one another or with other biological processes under specific conditions.

**Methods:**

We propose a novel method to standardize descriptions of enriched pathways for a set of genes/proteins using Gene Ontology terms. We used this method to examine the relationships among pathways and biological processes for a set of condition-specific genes/proteins, represented as a functional biological pathway-process network. We applied this algorithm to a set of 613 MI-specific proteins we previously identified.

**Results:**

A total of 96 pathways from Biocarta, KEGG, and Reactome, and 448 Gene Ontology Biological Processes were enriched with these 613 proteins. The pathways were represented as Boolean functions of biological processes, delivering an interactive scheme to organize enriched information with an emphasis on involvement of biological processes in pathways. We extracted a network focusing on MI to demonstrate that tyrosine phosphorylation of Signal Transducer and Activator of Transcription (STAT) protein, positive regulation of collagen metabolic process, coagulation, and positive/negative regulation of blood coagulation have immediate impacts on the MI response.

**Conclusions:**

Our method organized biological processes and pathways in an unbiased approach to provide an intuitive way to identify biological properties of pathways under specific conditions. Pathways from different databases have similar descriptions yet diverse biological processes, indicating variation in their ability to share similar functional characteristics. The coverages of pathways can be expanded with the incorporation of more biological processes, predicting involvement of protein members in pathways. Further, detailed analyses of the functional biological pathway-process network will allow researchers and scientists to explore critical routes in biological systems in the progression of disease.

## Background

The emergence of publicly available pathway databases has provided biologists excellent resources to attain a deeper understanding of biological mechanisms by providing organization to a large list of differentially expressed genes and proteins. Knowledge of molecular-level interactions and reactions has been curated in many knowledge databases, forming biological pathways. These knowledge databases include BioCarta (http://biocarta.com/), Kyoto Encyclopedia of Genes and Genomes (KEGG), Reactome, Protein Analysis Through Evolutionary Relationships (PANTHER), and MetaCyc [1-5]. Most often, pathways are organized as directed graphs of interacting molecules and often are accompanied by visualizations that demonstrate relationships among gene products, gene function types (e.g., regulation, activation, and inhibition) and translated protein locations (e.g., extracellular matrix, cell membrane, or nucleus). Recently, the integration of various omics data such as proteomics, genomics, transcriptomics, and metabolomics for knowledge discovery has drawn much attention [6-9]. In addition to the aforementioned pathway knowledge databases, the Gene Ontology (GO) Consortium pursues approaches to standardize the representation of gene products across different species and databases [10]. GO consists of a controlled vocabulary of terms, covering three domains: cellular components, molecular functions and biological processes. A GO Biological Process (GOBP) is a series of molecular events, with a defined beginning and end. However, a biological process is not equivalent to a pathway; GOBPs are assumed to be independent and do not represent the interactions among molecules.

Despite manual curation and careful revision, different knowledge databases could have different descriptions, participating molecules, interacting diagrams, and supporting literature for similar pathways. For example, considering the Transforming Growth Factors Beta (TGF-beta) signaling pathway in human, KEGG reported as *hsa04350: TGF-beta signaling pathway*, Reactome reported as *REACT_111102.4: Signaling by TGF-beta Receptor Complex*, and Biocarta reported as *h_tgfbpathway*. In detail, KEGG annotated 80 genes/proteins, Reactome annotated 120 genes/proteins, and Biocarta annotated 17 genes/proteins with TGF-beta signaling pathway. Descriptions of TGF-beta signaling pathway in the nucleus were excerpted to show related yet distinctive contents among KEGG, Reactome and Biocarta databases (Material in quote marks and italic type represents verbatim quotation from the knowledge databases):

**KEGG **- "*Once phosphorylated, R-Smads associate with the co-mediator Smad, Smad4, and the heteromeric complex then translocates into the nucleus. In the nucleus, Smad complexes activate specific genes through cooperative interactions with other DNA-binding and coactivator (or co-repressor) proteins*".

(http://www.genome.jp/kegg-bin/show_pathway?hsa04350)

**Reactome **- "*The general signaling scheme is rather simple: upon binding of a ligand, an activated plasma membrane receptor complex is formed, which passes on the signal towards the nucleus through a phosphorylated receptor SMAD (R-SMAD). In the nucleus, the activated R-SMAD promotes transcription in complex with a closely related helper molecule termed Co-SMAD (SMAD4)*".

(http://www.reactome.org/PathwayBrowser/#DIAGRAM = 170834&PATH = 162582)

**Biocarta **- "*The activated TGF-beta R1 phosphorylates SMAD2 and SMAD3, which bind to the SMAD4 mediator to move into the nucleus and form complexes that regulate transcription. SMADs regulate transcription in several ways, including binding to DNA, interacting with other transcription factors, and interacting with transcription corepressors and coactivators like p300 and CBP*".

(http://www.biocarta.com/pathfiles/h_tgfbpathway.asp).

These variations in knowledge representation among different databases prompt an urgent need for standard pathway representations. For a set of proteins or genes with enriched pathways and GOBPs, we propose a method that integrates molecular interaction, biological pathways and GOBP to standardize descriptions of pathways using GOBPs through the establishment of the functional biological pathway-process network. We demonstrated with the set of 613 proteins related to myocardial infarction (MI) from the MI-specific protein-protein interaction network [11].

## Methods

In this study, we started with 613 MI-specific proteins to find enriched pathways and GOBPs [11]. We performed analyses to statistically examine the similarities between pathways and biological processes and identify the hierarchical structures for the GOBPs. Based on the similarity score matrix and the structure of GOBPs, we established the logical circuitry between GOBPs and pathways, and visualize the circuitry with networks.

### Selection of condition-specific genes/proteins

We previously identified 613 proteins specific to MI in an MI-specific protein-protein interaction network (MIPIN); the network and its protein members were used here to demonstrate the developed method [11].

### Functional annotation analysis

Many tools are available to provide gene-annotation enrichment analysis and pathway mapping. We performed functional annotation analysis using DAVID Functional Annotation Tool, with the parameters *Count *to be 2 and *EASE *to be 0.05, to obtain enriched GOBP terms, KEGG and Reactome pathways [12].

### Statistical measure of inter-annotator agreement

We evaluated the pairwise similarity between different annotation terms, including GO terms and pathways using Kappa statistics because annotation terms sharing common members might be related to one another [13]. Considering a set of all annotated genes/proteins *G*, two annotation terms *T_i _*and *T_j _*annotated by two set of genes *G_i _*and *G_j _*(*i*≠*j*; *i,j *= 1, 2, ..., *N*), we denoted the number of proteins annotated by both terms as *a_ij_*, the number of proteins annotated by *T_i _*but not *T_j _*as *b_ij_*, the number of proteins annotated by *T_j _*but not *T_i _*as *c_ij_*, and the number of proteins not annotated by neither terms among the union of proteins annotated by *N *annotation terms as *d_ij_*.

Thus, we have,

$$G=\underset{N}{⋃}G_{i},a_{ij}=G_{i}\cap G_{j},b_{ij}=G_{i}\backslash G_{j},c_{ij}=G_{j}\backslash G_{i},d_{ij}=G\backslash \left(G_{i}\cup G_{j}\right)$$

The Kappa score *κ_ij _*was defined as,

$$\kappa_{ij}=\frac{\text{Pr}\left(agree_{ij}\right)-\text{Pr}\left(random_{ij}\right)}{1-\text{Pr}\left(random_{ij}\right)},$$

where Pr(*agree_ij_*) was the observed percentage agreement and Pr(*random_ij_*) was the overall probability of random agreement for annotation terms *T_i _*and *T_j_*. The observed percentage agreement Pr(*agree_ij_*) could be calculated as follows,

$$P\left(agree_{ij}\right)=\frac{a_{ij}+d_{ij}}{a_{ij}+b_{ij}+c_{ij}+d_{ij}}.$$

Out of total number of associated proteins, *T_i _*annotates $\left(a_{ij}+b_{ij}\right)/\left(a_{ij}+b_{ij}+c_{ij}+d_{ij}\right)$ and *T_j _*annotates $\left(a_{ij}+c_{ij}\right)/\left(a_{ij}+b_{ij}+c_{ij}+d_{ij}\right).$ Thus, the probability that both annotation terms randomly annotate the same proteins was$\left(a_{ij}+b_{ij}\right)\left(a_{ij}+c_{ij}\right)/{\left(a_{ij}+b_{ij}+c_{ij}+d_{ij}\right)}^{2}.$Similarly, the probability that neither pathway randomly annotate the same protein was $\left(b_{ij}+d_{ij}\right)\left(c_{ij}+d_{ij}\right)/{\left(a_{ij}+b_{ij}+c_{ij}+d_{ij}\right)}^{2}.$As a result, the overall probability of random agreement Pr(*random_ij_*) could be calculated as,

$$P\left(random_{ij}\right)=\frac{\left(a_{ij}+b_{ij}\right)\left(a_{ij}+c_{ij}\right)+\left(b_{ij}+d_{ij}\right)\left(c_{ij}+d_{ij}\right)}{{\left(a_{ij}+b_{ij}+c_{ij}+d_{ij}\right)}^{2}}.$$

A high Kappa score indicated that two annotation terms share many common proteins.

### Construction of undirected GOBP graph

An undirected GOBP graph *GraphGOBPenriched *was constructed to describe the relationships among *N_enrichedGOBP _*enriched GOBP terms, i.e., *GraphGOBPenriched *= (*V_GOBP_, E_GOBP_*), |*V_GOBP_|= N_enrichedGOBP_*, and *E_GOBP _*defines the set of edges in the graph. The relationships between GOBP terms, represented by edges connecting them, were evaluated based on the ancestor/offspring relationships in the complete directed acyclic graph of all GOBP terms from the Gene Ontology Consortium. We mapped *N_enrichedGOBP _*enriched GOBP terms to the corresponding vertices of the complete directed acyclic graph of all GOBP terms from the Gene Ontology Consortium using the package "GO.db" from Bioconductor [14]. Let *GraphGOBPComplete *= (*V_completeGOBP_, E_completeGOBP_*) be the complete directed acyclic graph of all GOBP terms. Then, *V_GOBP _*is mapped to *V_completeGOBP _*(*V_GOBP _*⊂ *V' *and *V' *⊂ *V_completeGOBP_*). Two GOBP terms would be connected if there existed a link between this pair of vertices in the complete graph of GOBP. All networks and graphs in this study were constructed and analyzed with the assistance of the package 'igraph' in R [15].

### Construction of undirected Boolean bipartite pathway and GOBP graph

**Figure 1:**
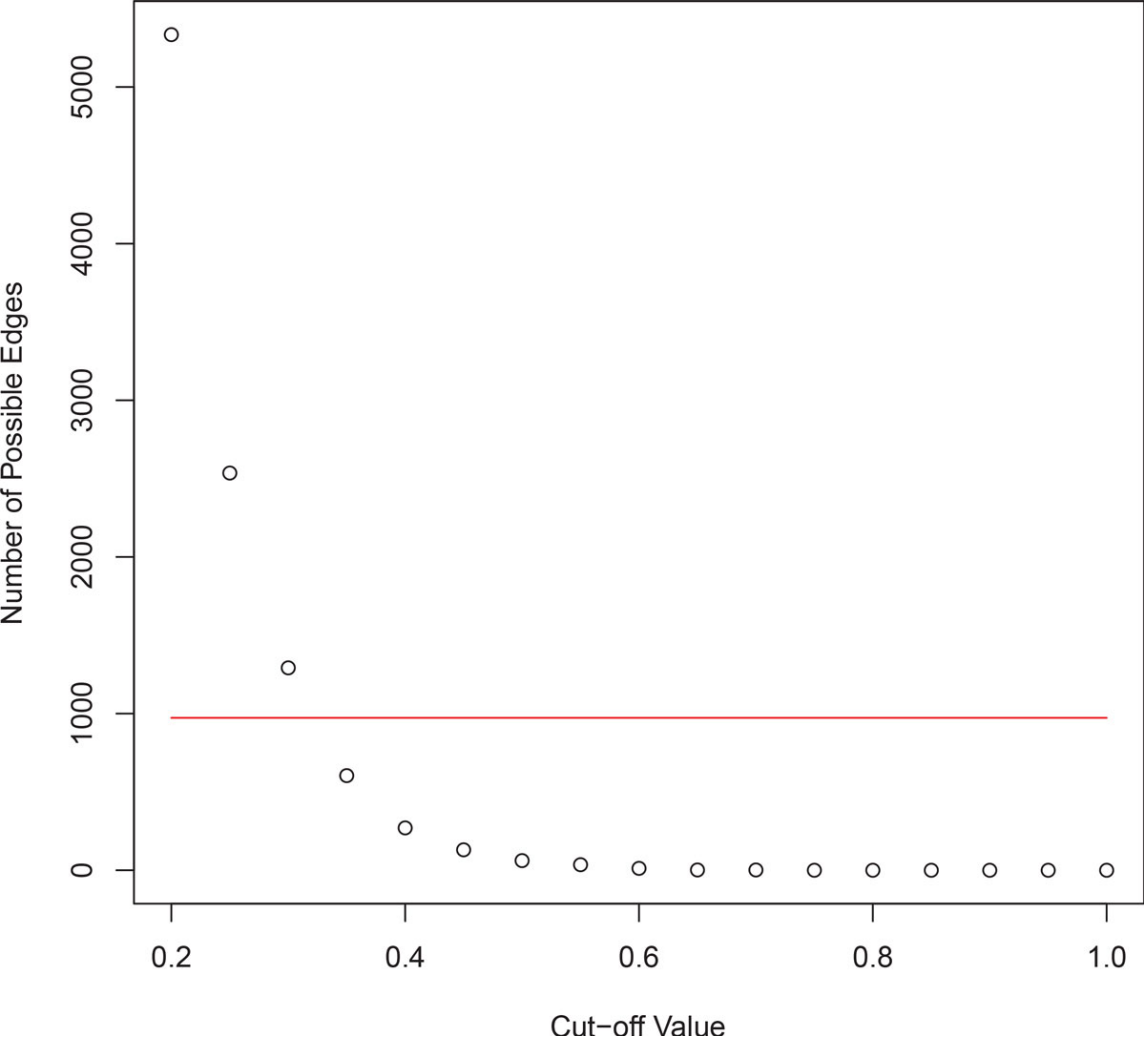
**The graph showing Number of possible edges vs. Cut-off value, and the selected number of edges**. Choosing top 1% of the most similar pairs of pathway and biological process considered a reasonable number of pairs of pathways and biological processes with high similarity scores.

The relationships between pathways and GOBP terms were represented as an undirected graph where edges between pathways and GOBP terms were evaluated based on Kappa statistics. We computed the Kappa similarity matrix of size *N_totalGOBP _*x *N_totalPathway_*, where *N_totalPathway _*is the total number of pathways including Biocarta, KEGG and Reactome pathways. Each row of the similarity matrix represents a GOBP term, and each column represents a pathway. Top 1% of the most similar pairs of pathway and biological process were selected and connected based on the Kappa similarity scores. Figure 1 showed that choosing the top 1% of the most similar pairs allowed the selection of a reasonable number of edges with high similarity scores (the average of Kappa scores was 0.025, and the chose cut-off value was 0.27). The set of pairs of pathway and GOBP terms satisfying such condition as was denoted as *E_PathwayGOBP_*. We then established the pathway and GOBP graph as an undirected bipartite graph *BipartiteGraphPathwayGOBP *= {*V_Pathway_, V_GOBP_, E_PathwayGOBP_*} where *V_Pathway _*is the set of pathways and *V_GOBP _*is the set of GOBP terms included in *E_PathwayGOBP _*(|*V_Pathway_| *≤ *N_totalPathway _*and |*V_B_*| ≤ *N_totalGOBP_*). Thus, the graph *BipartiteGraphPathwayGOBP *would consist of pathways that could be well represented by GOBP terms.

We further introduced Boolean rules to *BipartiteGraphPathwayGOBP *to represent pathways as Boolean functions of biological processes, assuming that connected biological processes have direct impacts on the pathways. Since a pathway contains dynamics and dependencies among participating molecules, which are annotated by biological processes, we assume that different combinations of biological process states can affect the state of the pathway, which is either 'active' (binary state 1) or 'inactive' (binary state 0). For every pathway *V_Pathwayi _*in the graph *BipartiteGraphPathwayGOBP*, let *V_PathwayGOBPi _*be the set of GOBP terms connected to that pathway and *V_GOBP _*= ∪*V_PathwayGOBPi-_*, we performed Boolean mapping such that the pathway *V_Pathwayi _*could be described as a Boolean algebra functions of its connected GOBP terms, *V_Pathwayi _*= *f*(*V_PathwayGOBPi_*).

The Boolean rules were derived from the relationships between GOBP terms connected to the pathway. If two GOBP terms were connected, then the Boolean relationship between these GOBP terms would be "OR." Such assumption arose from the fact two connected GOBP terms would share a significant amount of protein; thus, if a biological process was active, then its connected process must be simultaneously active as well. The relationship between two unconnected GOBP terms would be "AND." For example, considering a small network with 3 GOBP terms, *GOBP_1_, GOBP_2 _*and *GOBP_3_*, and a pathway *P*, where *GOBP_1 _*and *GOBP_2 _*were connected, *GOPB_3 _*was not connected with *GOBP_1 _*and *GOBP_2_*, and all GOBP terms were connected to pathway *P*. Then, the Boolean function for P could be written as, *V_P _*= (*V_GOBP1 _*∪ *V_GOBP2_) *∩ *V_GOBP3_*.

### The functional biological pathway-process network and the extracted MI network

We combined the GOBP graph *GraphGOBPenriched *from section 2.4 and the bipartite graph *BipartiteGraphPathwayGOBP *from section 2.5 to have a complete functional biological pathway-process network, where there were connections among GOBPs, and pathways communicated with each other through biological processes. As the complete network had many vertices and edges, we presented the MI pathway, *h_amiPathway*, from Biocarta, to illustrate the result. We retained important GOBP terms which were crossed by the shortest paths among other pathways to the MI pathway. Shortest paths were calculated using the un-weighted breadth-first search method. The extracted network allowed us to identify how the MI pathway could lead to other pathways and vice versa, initiating cardiac remodeling post-MI.

## Results

### Undirected GOBP graph

Using DAVID Functional Annotation Tool, we obtained 993 enriched GOBP terms from the list of 613 MI-specific proteins. From the ancestor/offspring relationships, the graph *GraphGOBPenriched *was constructed, resulting in a network of 993 vertices and 4284 edges. *GraphGOBPenriched *had 16 connected sub-graphs having more than 1 vertex and 46 isolated vertices. The largest connected sub-graph consisted of 885 vertices and 4199 edges.

It is interesting to note that GOBP terms with the highest degree, measuring the number of direct links incident on a vertex in a graph, were related to phosphorylation, phosphate, phosphorus, and kinase activity (Table 1). Since phosphorus and phosphate metabolic processes have the highest connections, this could mean that the chemical reactions and pathways involving intracellular signaling might initiate the cascade of events post-MI. In fact, serum phosphorus has been shown to serve as a sensitive indicator of MI and is linked to all-cause mortality and heart failure in patients after MI [16,17]. Hypophosphatemia in MI is associated with a greater degree of dysfunction of the left ventricle (LV), resulting in increased 30 days mortality [18]. In patients with MI, plasma sphingosine-1-phosphate concentration is reduced, leading to decreases protective action on cardiomyocyte viability [19].

**Table 1:** Top 20 GO Biological Processes ranked by degree measurements.

GOBP ID	Name	Degree
GO:0006793	phosphorus metabolic process*	55
GO:0006796	phosphate metabolic process*	52
GO:0006955	immune response	47
GO:0010033	response to organic substance	47
GO:0016310	phosphorylation*	45
GO:0048584	positive regulation of response to stimulus	45
GO:0051174	regulation of phosphorus metabolic process*	44
GO:0019220	regulation of phosphate metabolic process*	43
GO:0043507	positive regulation of JUN kinase activity*	43
GO:0042325	regulation of phosphorylation*	39
GO:0043406	positive regulation of MAP kinase activity*	38
GO:0000187	activation of MAPK activity*	38
GO:0032268	regulation of cellular protein metabolic process	37
GO:0031659	positive regulation of cyclin-dependent protein kinase activity during G1/S*	37
GO:0006468	protein amino acid phosphorylation*	36
GO:0010604	positive regulation of macromolecule metabolic process	34
GO:0001932	regulation of protein amino acid phosphorylation*	34
GO:0001775	cell activation	34
GO:0045860	positive regulation of protein kinase activity*	33
GO:0006952	defense response	33

*GOBPs related with phosphorus, phosphate, phosphorylation, and kinase activity.

In addition, biological processes involved with phosphorylation accounted for 4 GOBP terms while there were 5 kinase-activity-related GOBPs in Table 1. Phosphorylation is a major post-translational modification to regulate protein function. In a phosphorylation process, a protein kinase modifies target proteins, or substrates, by chemically adding phosphate groups to them. This result corresponded well with our previous work which identified Kinase Pathways as one of the major groups of pathways significantly enriched following MI [11].

### Network of biological pathways and GOBP showed similarities and differences among pathways in regard to GOBP annotation

At selected parameters, we retrieved 98 pathways, including 37 KEGG, 13 Reactome, and 48 Biocarta pathways using DAVID Functional Annotation Tool. Analysing statistical measures of inter-annotator agreement between 98 pathways and 993 GOBP terms, we established a graph *BipartiteGraphPathwayGOBP *with 544 vertices, containing 96 pathways 448 associated GOBPs, and 973 edges. These edges represented the most significantly enriched pairs of pathways and GOBP in the context of MI. This graph consisted of 8 sub-graphs, with the largest connected component having 76 pathways and 396 GOBP terms.

**Figure 2:**
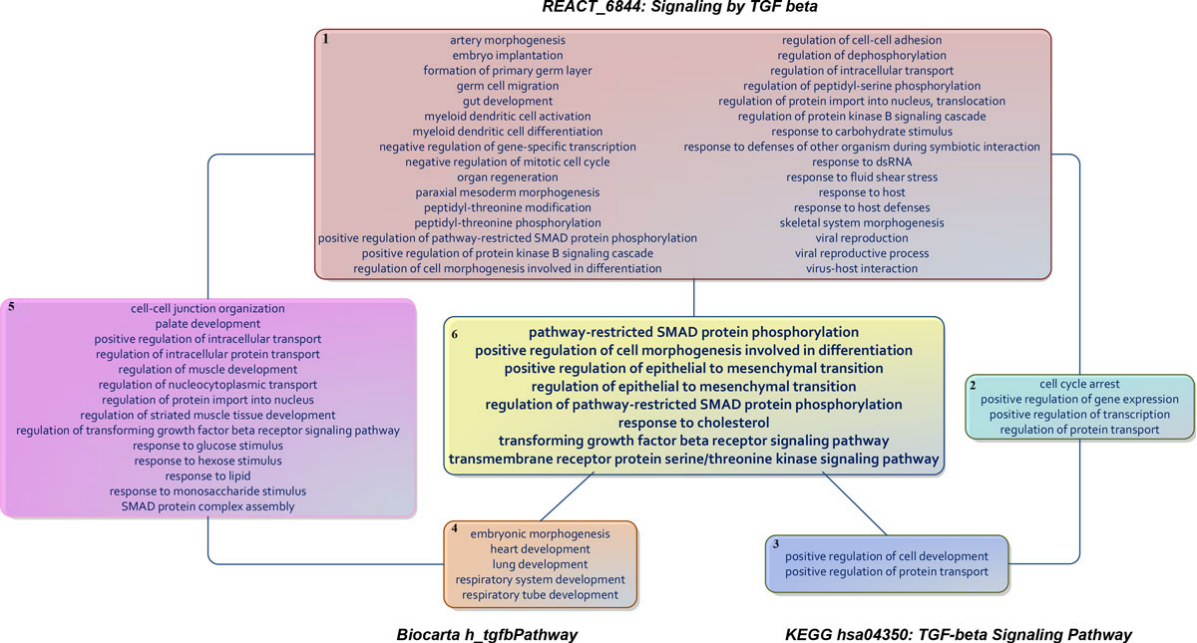
**Representations of TGF-beta signalling pathway from Biocarta, KEGG and RACTOME in terms of Gene Ontology biological processes in the condition of MI**. Box 1: GOBP exclusive to REACTOME ***REACT_6844: Signaling by TGF beta***. Box 2: Common GOBP between REACTOME and KEGG. Box 3: GOBP exclusive to KEGG ***has04350: TGF-beta Signaling Pathway***. Box 4: GOBP exclusive to BioCarta ***h_tgfbPathway***. Box 5: Common GOBP between BioCarta and REACTOME. Box 6: Common GOBP between BioCarta, KEGG and REACTOME.

Earlier, we mentioned the TGF-beta signaling pathway and how it was defined differently among the KEGG, Reactome, and Biocarta pathway databases. We further examined the associated GOBP terms to compare these 3 pathways (Figure 2). The variations were due to different literature being used to construct the pathways: *REACT_6844: Signaling by TGF beta *were involved with 56 GOBP terms, *hsa04350: TGF-beta Signaling Pathway *was associated with 14 GOBP terms, and the *h_tgfbPathway *was linked to 27 GOBP terms. Nonetheless, the common biological processes among these pathways included phosphorylation of SMAD proteins, serine/threonine kinase signaling pathway, epithelial-mesenchymal transition, and response to cholesterol and cell morphogenesis involved in differentiation (Figure 2: Box 6). It can be seen that the *REACT_6844 *provided a more complete description of TGFβ signaling pathway (Figure 2: Box 1-2&5), *hsa04350 *mainly focused on protein transport, transcription, gene expression and cell development (Figure 2: Box 2-3), whereas *h_tgfbPathway *emphasized organ development (Figure 2: Box 4-5). As a result, we can understand the different characteristics assigned for each pathway under the different circumstances. Individually, TGF-beta signaling pathways from KEGG, Biocarta, and Reactome annotated 21, 12, and 7 proteins, respectively, from the initial 613 MI-specific proteins. Thus, by incorporating the signaling pathways from different sources, we updated the knowledge of TGF-beta signaling pathways with more biological processes, and identified additional proteins participating in the pathway. Using this approach, the total number of proteins annotated with TGF-beta signaling pathways, by combining proteins from KEGG, Biocarta and Reactome, was expanded to 25 proteins.

**Figure 3:**
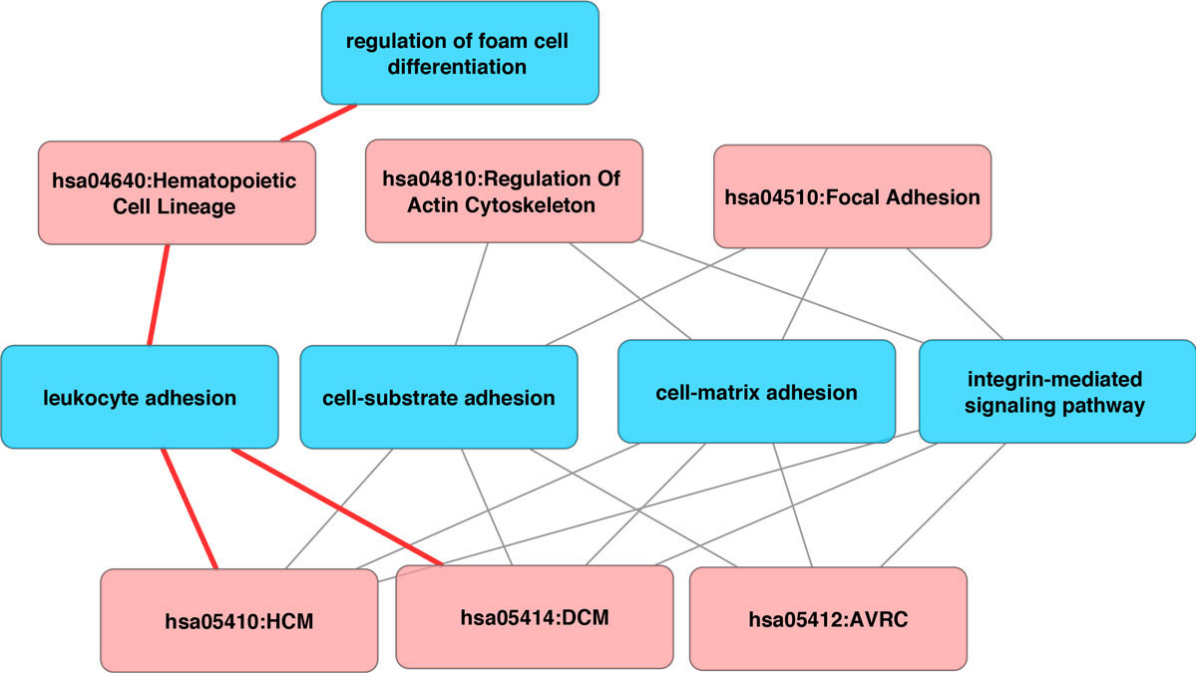
**Sub-network of Cardiomyopathy**. Pathways were represented in red while GOBPs were represented in blue. Pathways of Hypertrophic, Dilated and Arrhythmogenic Right Ventricular Cardiomyopathy were shown to be connected to biological processes including leukocyte adhesion, cell-substrated adhesion, and cell-matrix adhesion. Integrin-ECM interactions are required for cell adhesion.

Additionally, we investigated how this system acts using three other cardiovascular disease processes, namely *hsa05412: Arrhythmogenic Right Ventricular Cardiomyopathy *(ARVC), *hsa05410: Hypertrophic Cardiomyopathy *(HCM), and *hsa05414: Dilated Cardiomyopathy *(DCM). These analyses provide additional examples to demonstrate how representing pathways in terms of biological processes helped us to quickly understand the characteristics of such conditions under specific circumstances (Figure 3). ARVC is an inherited disease that results in fat and fibrous tissues replacing the heart muscle of the right ventricle and subepicardial region of the left ventricle. With HCM, a portion of the myocardium is hypertrophied, forcing the heart to work harder to pump blood because of the thickened heart muscle. DCM is a condition in which the heart weakens and becomes dilated, resulting in inefficient blood pumping to other organs. All three aforementioned cardiomyopathy pathways involve integrin-mediated signaling pathway, cell-matrix adhesion, and cell-substrate adhesion. However, HCM and DCM are specifically related to leukocyte adhesion. It has been confirmed that human leukocyte antigens are associated with HCM and DCM [20-23].

**Figure 4:**
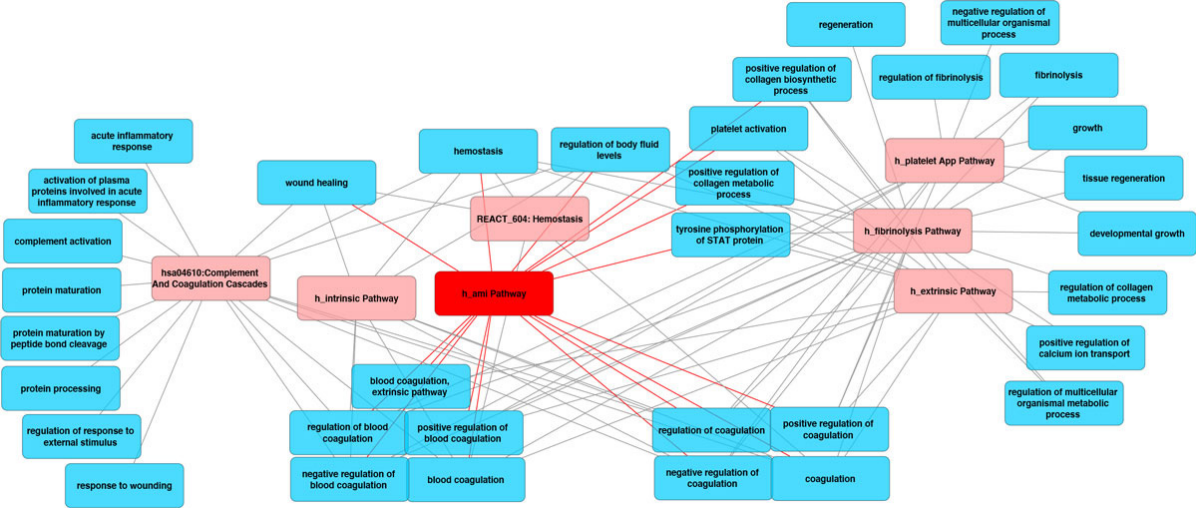
**Sub-network of MI**. Pathways were represented in while GOBPs were represented in blue. The major underlying processes for MI included coagulation, homeostasis, collagen metabolic/biosynthetic process, calcium ion transport, tissue regeneration, and wound healing.

We showed a visualization of a sub-graph consisting of 7 pathways and 34 GOBP terms that intersected with the MI response (Figure 4). Two pathways having the largest number of associated GOBP terms were *hsa04610: Complement And Coagulation Cascades *(characterized by 17 GOBP terms) and *h_fibrinolysisPathway *(characterized by 22 GOBP terms). The center of this sub-network is the MI pathway from Biocarta, *h_amiPathway*. Altogether, 3 pathways were represented by 32 out of 34 GOBP terms in this sub-network, and there were 8 common GOBP terms, including *coagulation, regulation of coagulation, negative regulation of coagulation, blood coagulation, regulation of blood coagulation, negative regulation of blood coagulation, homeostasis *and *regulation of body fluid levels *(Table 2). As a result, we noticed that blood coagulation, coagulation, homeostasis and regulation of body fluid levels were the underlying processes in these pathways. Table 2 and Figure 4 also pointed out the differences among these pathways: *hsa04610 *was associated with activation of proteins involved in acute inflammatory response and wound healing, whereas the fibrinolysis pathway was specifically involved with fibrinolysis, platelet activation, protein phosphorylation, collagen process and tissue regeneration.

**Table 2:** Pathways and GOBP in the MI functional pathway-process network.

GOBP names	Pathways	F	C	A	E	P	I	H
activation of plasma proteins involved in acute inflammatory response			√					
acute inflammatory response			√					
blood coagulation		√	√	√	√		√	√
blood coagulation, extrinsic pathway				√	√			
coagulation		√	√	√	√		√	√
complement activation			√					
developmental growth		√				√		
fibrinolysis		√				√		
growth		√				√		
hemostasis		√	√	√	√		√	√
negative regulation of blood coagulation		√	√	√		√	√	
negative regulation of coagulation		√	√	√		√	√	
negative regulation of multicellular organismal process						√		
platelet activation		√		√	√			
positive regulation of blood coagulation		√		√	√	√		
positive regulation of calcium ion transport		√			√			
positive regulation of coagulation		√		√	√	√		
positive regulation of collagen biosynthetic process		√		√	√			
positive regulation of collagen metabolic process		√		√	√			
protein maturation			√					
protein maturation by peptide bond cleavage			√					
protein processing			√					
regeneration		√						
regulation of blood coagulation		√	√	√		√	√	
regulation of body fluid levels		√	√	√	√		√	√
regulation of coagulation		√	√	√		√	√	
regulation of collagen metabolic process		√			√			
regulation of fibrinolysis						√		
regulation of multicellular organismal metabolic process		√			√			
regulation of response to external stimulus			√					
response to wounding			√					
tissue regeneration		√				√		
tyrosine phosphorylation of STAT protein		√		√	√			
wound healing			√	√			√	√
**Number of connected GOBPs**		**22**	**17**	**16**	**14**	**12**	**9**	**5**

### Associations between the MI response and biological processes have been experimentally and clinically verified

In order to confirm the affiliated biological processes with the MI response mentioned in the previous section, we searched PubMed for experimental and clinical evidence. In the *BipartiteGraphPathwayGOBP*, the MI pathway, annotated with 11 proteins, was connected with 16 GOBP terms that were linked to 64 proteins, and they shared 10 common proteins. We further verified that among the 54 proteins exclusively annotated by GOBP terms, 11 proteins had been chosen as the seed proteins to construct the MI-specific protein network. We have previously shown that these seed proteins were associated with MI and confirmed by at least 2 citations [11].

To verify that the remaining 43 proteins of the expanded set of proteins for the MI pathway were related to MI, we searched for their official names and aliases on PubMed along with the keyword "myocardial infarction" for publications that confirmed the association between these proteins and MI (Table 3). There were 34 proteins firmly associated with MI by at least 2 publications. There were 3 proteins, namely CD44, SERPIND1 and HNF4A, directly associated with MI by one publication. There were 6 proteins, namely ANXA7, FBLN5, FGF7, KLF6, FR2RL2 and GGCX indirectly linked to MI. Among 16 MI-associated GOBP terms, 11 biological processes were fully associated with the MI pathway as all of their member proteins were associated with MI and confirmed by at least 2 publications. The remaining 5 GOBP terms had 90% of the member proteins associated with the MI pathway, confirmed by at least 1 publication, and 80% or more of the member proteins were confirmed to be associated with MI by at least 2 publications. Therefore, we showed that the associations between MI pathway and biological processes in the *BipartiteGraphPathwayGOBP *have been experimentally and clinically verified. We also expanded the coverage of the original MI pathway by adding 54 new proteins. Further research will be needed to address the intermediate steps within the MI pathway and develop more extensive description of the MI pathway that covers a longer time scale.

**Table 3:** Proteins of MI pathway-associated GOBP terms with cited publications.

Proteins	Gene names	Official Names	Supporting Articles
A1AT_HUMAN	SERPINA1	Alpha-1-antitrypsin	[27,28]
ACVL1_HUMAN	ACVRL1	Serine/threonine-protein kinase receptor R3	[29,30]
ADA17_HUMAN	ADAM17	Disintegrin and metalloproteinase domain-containing protein 17	[31,32]
ANPRA_HUMAN	NPR1	Atrial natriuretic peptide receptor 1	[33,34]
APOA_HUMAN	LPA	Apolipoprotein(a)	[35,36]
CAV1_HUMAN	CAV1	Caveolin-1	[37,38]
CBPB2_HUMAN	CPB2	Carboxypeptidase B2	[39,40]
CD36_HUMAN	CD36	Platelet glycoprotein 4	[41,42]
EGLN_HUMAN	ENG	Endoglin	[29,30]
F13A_HUMAN	F13A1	Coagulation factor XIII A chain	[43,44]
FA11_HUMAN	F11	Coagulation factor XI	[45,46]
FA5_HUMAN	F5	Coagulation factor V	[47,48]
FA8_HUMAN	F8	Coagulation factor VIII	[43,45]
FA9_HUMAN	F9	Coagulation factor IX	[45,46]
FIBG_HUMAN	FGG	Fibrinogen gamma chain	[49,50]
FINC_HUMAN	FN1	Fibronectin	[51,52]
GPV_HUMAN	GP5	Platelet glycoprotein V	[53,54]
HIF1A_HUMAN	HIF1A	Hypoxia-inducible factor 1-alpha	[55,56]
IC1_HUMAN	SERPING1	Plasma protease C1 inhibitor	[57,58]
IFNG_HUMAN	IFNG	Interferon gamma	[59,60]
ITA5_HUMAN	ITGA5	Integrin alpha-5	[61,62]
KNG1_HUMAN	KNG1	Kininogen-1	[63,64]
LYOX_HUMAN	LOX	Protein-lysine 6-oxidase	[65,66]
PAR2_HUMAN	F2RL1	Proteinase-activated receptor 2	[67,68]
PAR4_HUMAN	F2RL3	Proteinase-activated receptor 4	[67,69]
PGFRA_HUMAN	PDGFRA	Platelet-derived growth factor receptor alpha	[70,71]
PLF4_HUMAN	PF4	Platelet factor 4	[72,73]
PROZ_HUMAN	PROZ	Vitamin K-dependent protein Z	[74,75]
SMAD3_HUMAN	SMAD3	Mothers against decapentaplegic homolog 3	[30,76]
TGFB2_HUMAN	TGFB2	Transforming growth factor beta-2	[77,78]
TGFR2_HUMAN	TGFBR2	TGF-beta receptor type-2	[79,80]
TRBM_HUMAN	THBD	Thrombomodulin	[81,82]
TSP1_HUMAN	THBS1	Thrombospondin-1	[83,84]
UROK_HUMAN	PLAU	Urokinase-type plasminogen activator	[85,86]
CD44_HUMAN	CD44	CD44 antigen	[87]
HEP2_HUMAN	SERPIND1	Heparin cofactor 2	[88]
HNF4A_HUMAN	HNF4A	Hepatocyte nuclear factor 4-alpha	[89]
ANXA7_HUMAN	ANXA7	Annexin A7	[90]
FBLN5_HUMAN	FBLN5	Fibulin 5	[91]
FGF7_HUMAN	FGF7	Fibroblast growth factor 7	[92]
KLF6_HUMAN	KLF6	Krueppel-like factor 6	[93]
PAR3_HUMAN	F2RL2	Proteinase-activated receptor 3	[94]
VKGC_HUMAN	GGCX	Vitamin K-dependent gamma-carboxylase	[95]

Proteins with indirect association with MI were contained in shaded box.

### Phosphorylation of STAT protein, coagulation and regulation of collagen process are required to activate the MI pathway

**Figure 5:**
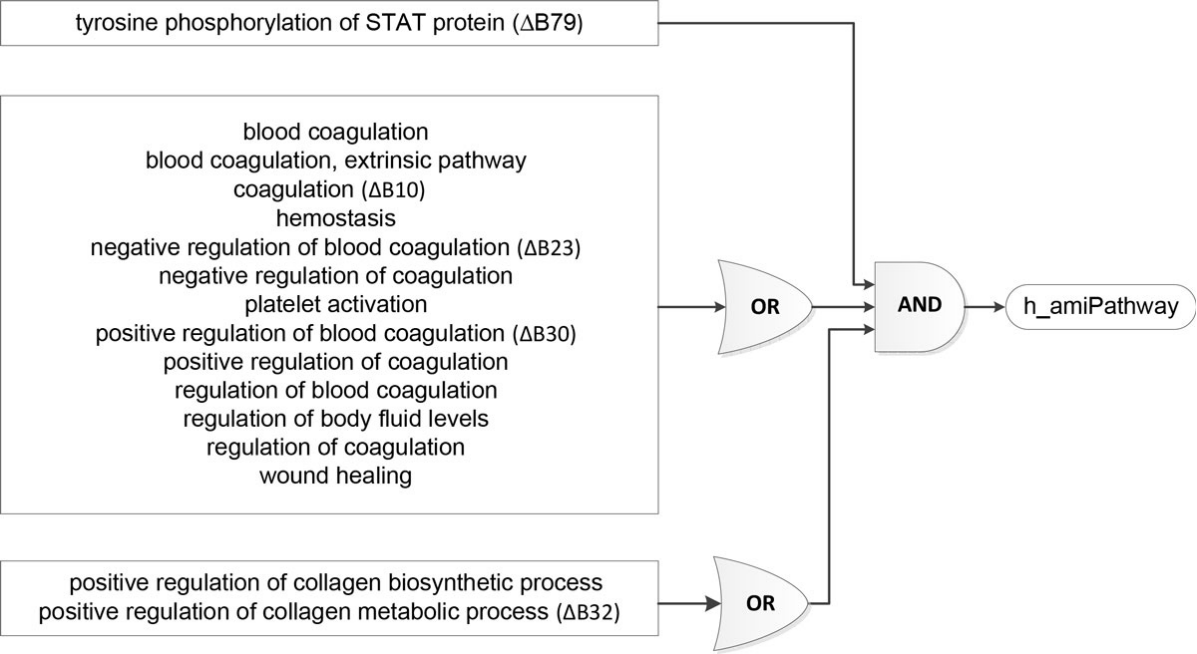
**Logical circuit of *h_amiPathway***. Logical circuits described the relationships between GO biological processes and the MI pathway. We used multiple input single output logical gates AND and OR, where the GOBP were the inputs and ***h_amiPathway ***were the outputs. The extracted network of MI identified five major GOBP terms, including tyrosine phosphorylation of STAT protein (ΔB79), coagulation (ΔB10), negative and positive regulation of blood coagulation (ΔB23 & ΔB30), and positive regulation of collagen metabolic process (ΔB32), required to activate the MI pathway. The labels next to the name of the GOBP terms corresponded to the legend in Figure 6.

**Figure 6:**
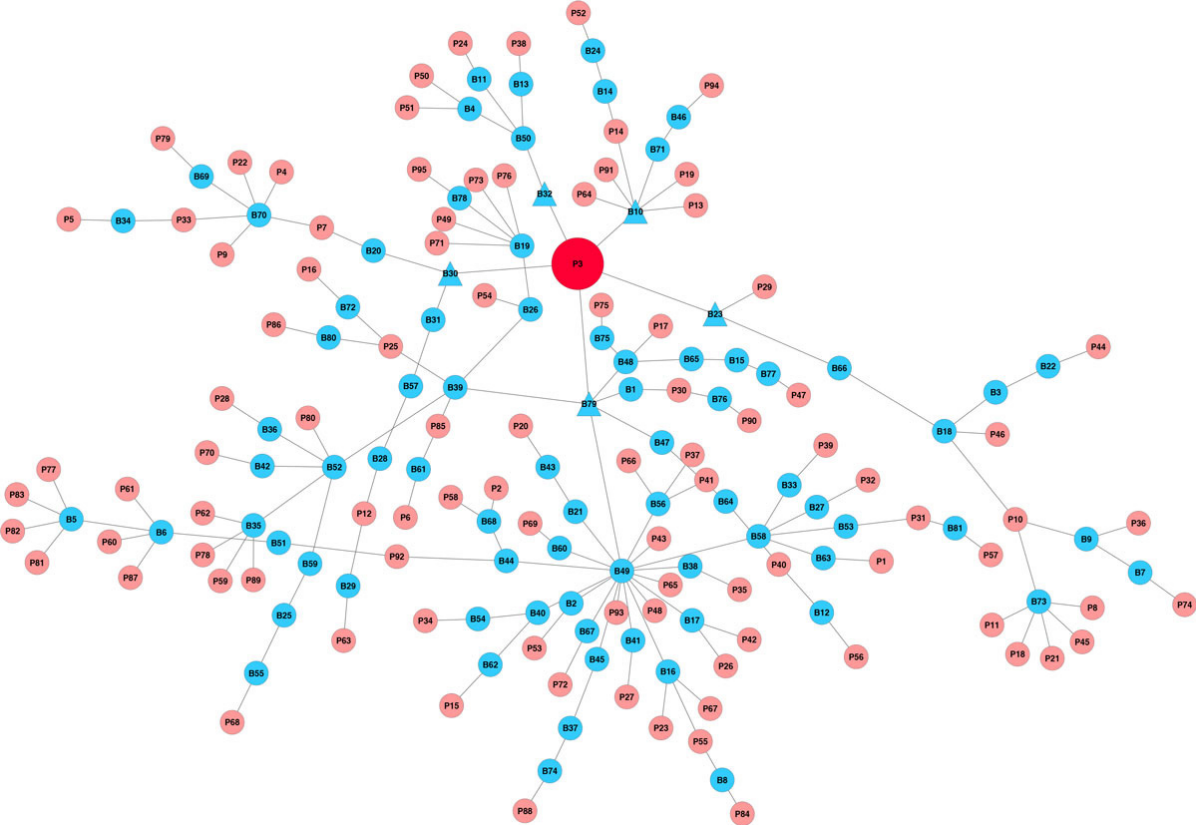
**The extracted MI network**. The acute MI pathway was colored in red while other pathways were colored in light red. Biological processes were represented in blue circles. GOBPs having direct impact on h_amiPathways were represented as blue triangles. A small branch of the network inside the blue rectangle involving coagulation was zoomed out for demonstration. Below are legends for selected pathways and processes (for the complete list of pathways and processes, see Supplemental Table 1). P3: h_amiPathway. P40: h_tgfbPathway. P58: hsa04350:TGF-betaSignalingPathway. P92: REACT_6844:Signaling by TGF beta. B10: coagulation. B30: positive regulation of blood coagulation. B32: positive regulation of collagen metabolic process. B44: positive regulation of protein kinase B signaling cascade. B58: regulation of kinase activity. B49: protein kinase cascade. B79: tyrosine phosphorylation of STAT protein.

We further explored the possibility of representing pathways as Boolean functions of biological processes. This idea originates from the fact that proteins within biological system typically act in concert. Biological processes are processed through protein-protein or molecular interactions, which usually have similar functions. The establishment of the bipartite graph of pathways and GOBP yielded Boolean functions to determine the state of pathways based on biological processes. We illustrated the MI pathway *h_amiPathway *as logic circuits with multiple input single output logic gates (Figure 5). The MI pathway requires tyrosine phosphorylation of STAT protein, either positive regulation of collagen biosynthetic process or metabolic process, and one or more of the GOBP terms in the large group for activation. We later extracted the MI network, and identified the five major GOBP terms that contributed to the activation of *h_amiPathway *(Figure 6; see Additional file 1 for names of all pathways and GOBPs in the MI network). Tyrosine phosphorylation of STAT protein, negative and positive regulation of blood coagulation, coagulation and positive regulation of collagen metabolic process are required to activate the MI pathway. By displaying pathways as logic circuits, we could observe the involvement of multiple functional groups, thus providing an intuitive way to understand associated pathways.

### Critical routes of the extracted MI network

The complete network of pathways and GOBP contains a huge amount of information although it could be overwhelming. We extracted the MI network and only retained the backbone to explore additional features that might not have been covered. Figure 6 showed the routes from MI-related pathways, represented as light red circles, to the *h_amiPathway*, whose color was in red, through biological processes in as blue circles. The graph was undirected, meaning some routes could be bidirectional. A small branch of the network inside the blue rectangle was zoomed out for illustration purpose. The complete list of pathways and GOBP can be viewed in Supplemental Table 1. We observed that all 7 pathways in those 6 branches needed to pass through coagulation to be connected to *h_amiPathway*. We found the cell cycle pathway, *hsa04110:CellCycle*, particularly interesting since the pathway was linked to *h_fibrinolysisPathway*, through *cell growth*. Heissig et. al (2007) showed that by deleting plasminogen, a classical fibrinolytic factor that controls hematopoietic stress response, in mice, hematopoietic stem cells were prevented from entering the cell cycle and undergoing multilineage differentiation after myelosuppression, leading to the death of the mice [24]. In other words, the plasminogen fibrinolytic pathway is crucial for hematopoietic regeneration. In another study, Heidt et al. (2014) showed that hematopoetic stem cells in the bone marrow could be activated by chronic stress, and further differentiated into increasing number of leukocytes. These leukocytes travel into the blood circulation and participate in the development of cardiovascular diseases [25]. Incidentally, fibrinolytic therapies have been used to enhance restoration of myocardial flow in the epcicardial infarct-related coronary artery [26]. Thus, it will be interesting to investigate the role of fibrinolysis and the increasing number of leukocytes in the cardiac remodelling post-MI and heart failures.

## Discussion

In this study, we established a network by integrating GO biological processes and pathways from BioCarta, KEGG, and REACTOME enriched for MI-specific proteins using statistical measures and hierarchical structures. We examined the similarities between pathways and biological processes, and derived Boolean models of pathways in terms of standardized vocabulary with GOBP terms. This network can be used to explore critical routes that connect pathways and biological processes to the development of diseases or conditions. To demonstrate a functional interaction network, we started from the proteins in an MI-specific protein-protein interaction network we had previously constructed, acquired the enriched GO biological processes and pathways, constructed the GOBP graph and the functional pathway-process network, and determined the logical circuitry representing the involvement of GOBPs in pathways. The approach could be used with any set of genes or proteins, specific to any conditions or diseases, to develop additional features and visualizations.

This study presented three important results. First, we established a MI-specific functional biological pathway-process network, with demonstrated sub-networks shown in Figures 2 and 3. We standardized pathway descriptions by their connected GOBP terms, making it easier to compare differences and similarities between pathways, especially those with similar descriptions from different databases. We provided an example in section 3.2 with TGF-beta signaling pathways and pointed out the common and exclusive biological properties from BioCarta, KEGG and REACTOME. Second, we derived the relationships between GOBP terms based on the hierarchical structure defined in the GO Consortium and organized these terms into functional groups that could contribute differently to the pathways. For each pathway, GOBP terms that belonged to different functional groups should act simultaneously to activate the pathway, whereas only one process in a functional group was needed initiate the function. We used multiple input single output logical gates AND and OR, where the GOBP were the inputs and pathways were the outputs. We built two logic circuits corresponding to the MI and fibrinolysis pathways. It was shown that tyrosine phosphorylation of STAT protein, coagulation and regulation of collagen process were required to activate the MI pathway. We also provided experimental and clinical evidence for the association between the MI pathway and biological processes. Third, we illustrated a centralized version of the complete network of pathways and GOBP, providing insights of critical routes from and to the main pathway, *h_amiPathway*. Because MI was the major theme of this study, this extracted network allowed us to quickly visualize the connection between pathways before and after MI and their involvement in the changes in the post-MI myocardium.

Our results illustrated that using the functional biological pathway-process network is a promising method to identify biological properties of pathways under specific conditions. Pathways having similar descriptions encompassed both similar and diverse biological processes, indicating variation in their ability to share similar functional characteristics. The coverages of biological pathways can be increased with the incorporation of more biological processes and protein members, promoting more comprehensive pathways. As we discover and understand more about genes and proteins, the network helps to expand the participating genes or proteins in the pathways through the introduction of related genes in the GOBP. Pathways will be more comprehensive, leading to better knowledge of diseases. However, functional groups of GOBP terms based on hierarchical structures might need to be further evaluated for coherence. Moreover, GOBP functional groups might not have the same amount of contribution to the corresponding pathways; probabilistic Boolean models would allow more robustness in the face of uncertainty. In conclusion, we report here the establishment of the network of pathways and biological processes that can be used as a foundation to identify biological properties of pathways, providing interaction and visualization of biological systems at pathway level.

## List of abbreviations used

AVRC: Arrhythmogenic Right Ventricular Cardiomyopathy

DCM: Dilated Cardiomyopathy

GO: Gene Ontology

GOBP: Gene Ontology Biological Process

HCM: Hypertrophic Cardiomyopathy

KEGG: Kyoto Encyclopaedia of Genes and Genomes

MI: Myocardial Infarction

MIPIN: Myocardial Infarction-Specific Protein-Protein Interaction Network

PANTHER: Protein Analysis Through Evolutionary Relationships

STAT: Signal Transducer and Activator of Transcription

TGF-beta: Transforming Growth Factor Beta

## Competing interests

The authors declare that they have no competing interests.

## Authors' contributions

Conceived and designed the experiments: NTN, MLL, and YFJ. Analyzed or reviewed the data: NTN, MLL, and YFJ. Contributed reagents/materials/analysis tools: NTN and YFJ. Wrote or edited the paper: NTN, MLL, and YFJ.

## Supplementary Material

Additional File 1 **Pathways and GOBPs of MI network**. This file contains names of pathways and GOBPs in the extracted network of MI with labels as displayed in Figure 6. Pathways were ordered alphabetically with prefix "P". GOBPs were ordered alphabetically with prefix "B". Click here for file

## Declarations

The publication costs for this article was funded by the corresponding author.

This article has been published as part of *BMC Genomics *Volume 16 Supplement 7, 2015: Selected articles from The International Conference on Intelligent Biology and Medicine (ICIBM) 2014: Genomics. The full contents of the supplement are available online at http://www.biomedcentral.com/bmcgenomics/supplements/16/S7.
